# Correction: Philic–phobic chemical dynamics of a 1^st^ tier dendrimer dispersed o/w nanoemulsion

**DOI:** 10.1039/d3ra90105j

**Published:** 2023-11-06

**Authors:** Naveen Kumari, Man Singh, Hari Om, K. M. Sachin

**Affiliations:** a Department of Chemistry, Deenbandhu Chhotu Ram University of Science and Technology Murthal Haryana India hariom.chem@dcrustm.org nkandhil90@gmail.com; b School of Chemical Sciences, Central University of Gujarat Gandhinagar Gujarat India mansingh50@hotmail.com sachinbbau@gmail.com

## Abstract

Correction for ‘Philic–phobic chemical dynamics of a 1^st^ tier dendrimer dispersed o/w nanoemulsion’ by Naveen Kumari *et al.*, *RSC Adv.*, 2019, **9**, 12507–12519, https://doi.org/10.1039/C9RA00728H.

In the original article, the authors regret errors in the mechanism proposed in Fig. 10, and the omission of the synthesis method used to synthesize the dendrimer ‘TTDMM’.

In the original article, in Fig. 10, the authors included a proposed and possible antioxidant free radical mechanism of TTDMM–oils (OO, CO and LO). An independent expert has confirmed that the proposed mechanism would not be possible with the current explanation and therefore needs further exploration. This does not affect the results or conclusions of the article.

In addition, in the original article, the synthesis procedure used for the synthesis of the dendrimer ‘TTDMM’ was not reported. The dendrimer ‘TTDMM’ sample used in the research was prepared according to the method described in ref. 1.

The Royal Society of Chemistry apologises for these errors and any consequent inconvenience to authors and readers.

## Supplementary Material
